# Pragmatism in practice: lessons learned during screening and enrollment for a randomised controlled trial in rural northern Ethiopia

**DOI:** 10.1186/s12874-018-0486-x

**Published:** 2018-03-07

**Authors:** Meseret Molla, Henok Negussie, Moses Ngari, Esther Kivaya, Patricia Njuguna, Fikre Enqueselassie, James A. Berkley, Gail Davey

**Affiliations:** 1 Centre for Environment and Development Studies, Addis Ababa University, Addis Ababa, Ethiopia; 2 Wellcome Trust Centre for Global Health Research, Brighton & Sussex Medical School, University of Sussex, Brighton, BN1 9PX UK; 3 KEMRI/Wellcome Trust Research Programme, Kilifi, Kenya; 4 School of Public Health, Addis Ababa University, Addis Ababa, Ethiopia; 5 Centre for Tropical Medicine & Global Health, University of Oxford, Oxford, UK

**Keywords:** External validity, Internal validity, Lessons, Pragmatic, Randomised controlled trial

## Abstract

**Background:**

We use the example of the Gojjam Lymphoedema Best Practice Trial (GoLBeT), a pragmatic trial in a remote rural setting in northern Ethiopia, to extract lessons relevant to other investigators balancing the demands of practicality and community acceptability with internal and external validity in clinical trials.

**Methods:**

We explain in detail the preparation for the trial, its setting in northern Ethiopia, the identification and selection of patients (inclusion and exclusion criterion, identifying and screening of patients at home, enrollment of patients at the health centres and health posts), and randomisation.

**Results:**

We describe the challenges met, together with strategies employed to overcome them.

**Conclusions:**

Examples given in the previous section are contextualised and general principles extracted where possible. We conclude that it is possible to conduct a trial that balances approaches that support internal validity (e.g. careful design of proformas, accurate case identification, control over data quality and high retention rates) with those that favour generalisability (e.g. ‘real world’ setting and low rates of exclusion). Strategies, such as Rapid Ethical Assessment, that increase researchers’ understanding of the study setting and inclusion of hard-to-reach participants are likely to have resource and time implications, but are vital in achieving an appropriate balance.

**Trial registration:**

ISRCTN67805210, registered 24/01/2013.

## Background

Randomized controlled trials (RCTs) are the cornerstone of evidence-based decision making and are considered the ‘gold standard’ for clinical research in both low- and high-income countries. In recent years, a movement towards health care decision-making that is more informed by evidence has boosted clinical trials [1]. RCTs are needed globally to reduce disease burdens by informing the development of safe and effective treatment and prevention strategies [2]. However, although developing countries bear the largest burden of disease and high incidence rates, few RCTs are conducted in such settings [3]. RCTs are critically important for developing countries, not just for their potential to improve health by establishing the true effectiveness of new interventions [2], but also for their potential to build health research capacity [4].

In resource limited countries like Ethiopia, the human and material resources available to ensure a high level of internal validity through design, management, and operation of clinical trials lag behind those of wealthier nations [2]. The low number of trials in such countries has also been attributed to issues such as difficulty obtaining meaningful informed consent [5] and balancing risks, benefits, compensation and the potential for unintended inducement among extremely poor populations in the context of poor health infrastructure and considerable socio-economic and cultural divides [6]. Many trials conducted in low income settings in the past were designed by experts based in higher-income countries because of a lack of trained personnel and expertise locally [7].

One important response to this lack of expertise is to draw lessons and disseminate experience from the few trials that are conducted in these settings. Here, we draw examples from ‘GoLBeT’ (the **Go**jjam **L**ymphoedema **Be**st Practice **T**rial), a pragmatic trial among podoconiosis patients in a remote rural setting in northern Ethiopia. Podoconiosis is a slowly progressive geochemical lymphoedema associated with long-term barefoot exposure to volcanic soils in tropical highland areas [11]. It results in significant social [12], physical [13] and economic [14] burdens to patients, and hinders development of affected communities. GoLBeT was designed to evaluate the effectiveness of a simple foot hygiene package in reducing acute dermatolymphangioadenitis (ADLA), one of the most common and disabling consequences of podoconiosis [13]. The results were intended to fill a gap in evidence and inform government policy in Ethiopia and potentially in other endemic countries. Pragmatic trials measure effectiveness of an intervention in routine practice [8], so external validity is a high priority. GoLBeT aimed to generate generalizable data with high utility for policy makers without significant comprise of internal validity [9]. Here, we explain in detail the preparation for the trial, its setting in northern Ethiopia, the identification and selection of patients (inclusion and exclusion criterion, identifying and screening of patients at home, enrollment of patients at the health centres and health posts), and randomisation. Through this, we aim to identify lessons that may be of use to others trying to balance internal and external validity [9] in pragmatic trials in similar settings.

## Methods

### Trial background and governance

GoLBeT was a pragmatic randomized controlled trial of podoconiosis lymphoedema management (ISRCTN67805210). The trial was funded by the UK Joint Global Health Trials initiative in 2013. The protocol, including numbers and dates of all ethical approvals, has been published [10]; in essence, the trial compared patients randomized to the intervention arm (who were trained to self-care using a hygiene and foot care intervention which comprised use of soap, Whitfield ointment, bandages, socks and shoes, exercise and elevation) with patients randomized to the control arm, who were followed quarterly but received no intervention until the trial was completed. The primary outcome was reduction in frequency of ADLA episodes. Patients in both groups filled in a monthly primary outcome diary and Case Report Forms (CRFs) were completed at 3-monthly intervals.

### Trial setting and participants

**Fig. 1:**
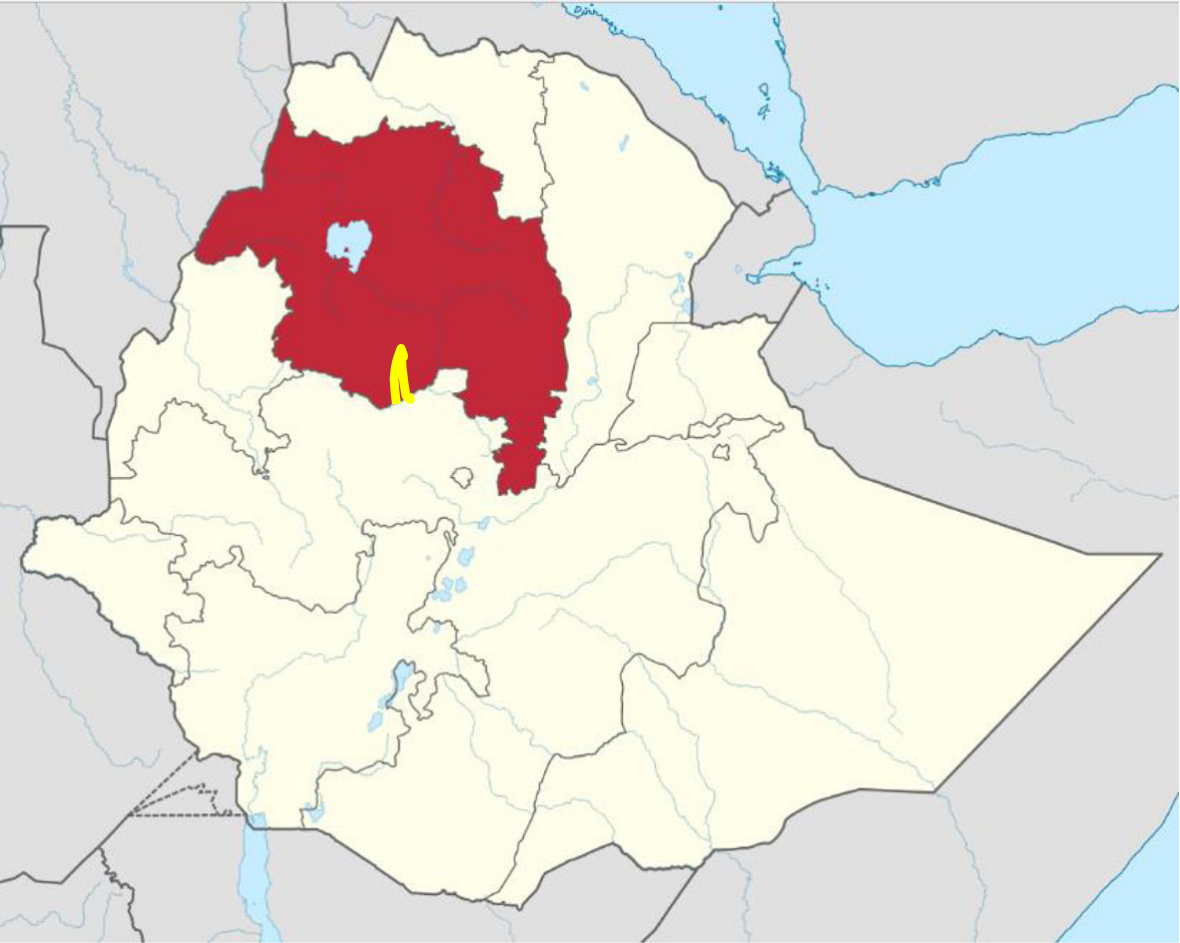
Map of Amhara Region, showing location of Aneded district

**Fig. 2:**
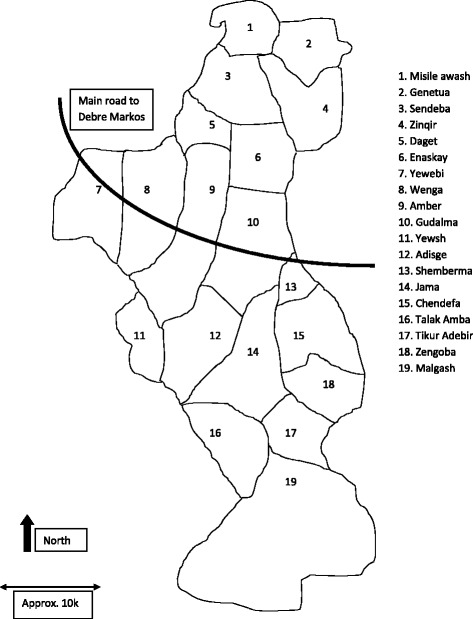
Map of Aneded district showing *kebeles*. Rounds 1 and 2 recruitment took place in *kebeles* 7–14 and 16, and round 3 in *kebeles* 1–6, 15, 17 and 18

The trial was conducted in Aneded district, East Gojam Zone, Amhara regional state (Fig. 1). There are 19 *kebeles* (sub-districts) in Aneded district (Fig. 2), which is 18 km from the zonal capital, Debre Markos, where the trial field coordination office was located [11]. Debre Markos is 305 km from Addis Ababa, a drive of approximately six hours which crosses the Blue Nile Gorge. At the time of this trial, no formal government or private treatment for podoconiosis was available within Aneded district. Within the district, there are two health centres, and each *kebele* has a health post. Reaching these health posts could take up to two hours from the asphalt in the dry season, and longer in the rainy season. We made the decision not to include the most remote *kebele* (*Malgash*, 19 in Fig. 2) even though many patients were registered there, because access entailed a 1.5 h drive and then a 1.5 h walk. However, patients in *Malgash* were given education about podoconiosis and three months’ treatment supplies, at the close of the trial.

Inclusion and exclusion criteria are detailed in the protocol [10], and their application resulted in exclusion of only 19 of 1339 patients screened.

### External support and monitoring

The trial coordinator and data manager spent two weeks at the KWTRP Clinical Trials Facility during the preparatory period. They were given hands-on training in several important aspects of trial management including development of Standard Operating Procedures (SOPs), logs, forms and master files; use of GPS; handling of the intervention product; Good Clinical Practice (GCP) training of field staff; management of hard-copy data; OpenClinica and other database development. Trial monitoring site visits were conducted by staff of the KWTRP Clinical Trials Facility and the Nuffield Department of Tropical Medicine, Oxford University. A Trial Preparation Visit was made in April 2013, a Site Initiation Visit in December 2014, Routine Monitoring Visits were made in March 2014 and December 2015, and a Close Out Visit in May 2017. These visits and a series of Skype calls aimed to review protocol fidelity and regulatory compliance.

### Development of trial Proformas

Under guidance from the KWTRP Clinical Trials Facility, SOPs were developed by the trial coordinator, the data manager (both based in Ethiopia) and the UK-based trial principal investigator through email exchanges, weekly Skype meetings and quarterly face-to-face meetings. Information gathered through a Rapid Ethical Assessment [12] was used to develop the patient information sheet and informed consent forms. Meetings to explain the trial were held with district Health Office personnel and with larger gatherings (including administrative, education, agriculture and religious leaders and Health Extension Workers from *kebeles* selected for the study). These aimed to disseminate accurate information about the trial through the district and to garner support of the various departments to ensure the smooth conduct of the trial.

CRFs were prepared for baseline, 3, 6, 9 and 12 months, according to the data collection schedule described in the protocol [10]. These were made easily distinguishable through different coloured covers (white, blue, green, purple and yellow, respectively). In addition, a patient-completed pictorial diary was developed. Patients were trained to complete the diary by inserting a mark in the appropriate column for that day – either one headed by a picture of a person in bed with ADLA or one headed by a person working in the field (both men and women work in the field in this part of rural Ethiopia). The diary was tested for acceptability, feasibility and accuracy using patient interviews and assessment of patient records against health professional diagnoses. The Ethiopian calendar was used for data collection and entry.

### Recruitment and training of field staff

Twenty Community Podoconiosis Agents (CPAs), and two CPA supervisors; ten data collectors and two data collector supervisors were recruited and given five days’ training on GCP, screening, informed consent administration and enrollment (all field staff); on data collection, data management and data security procedures (data collectors and their supervisors); and on intervention training, outcome diary checking and adherence monitoring (CPAs). Because of delays in gaining ethical approval, another five days’ refresher training was given immediately before enrollment commenced.

### Database development

After consideration of continuity of power supply, internet access, servicing, security and protection from water damage, the server was located in the IOCC office in Addis Ababa rather than in Debre Markos. An OpenClinica template was created and tested prior to data entry. Difficulties exporting data to other programmes were resolved. Two data entry clerks were given three days’ training to enter baseline data from the Case Report Forms. Access to the database from the KWTRP Clinical Trials Facility was tested several times before data entry was commenced.

### Ethical and regulatory considerations

Ethical approval required three serial applications. Approval from the School of Public Health was a prerequisite of applying to the Addis Ababa University Institutional Review Board (IRB). Approval from the IRB was necessary before the process could continue with the national regulator and the national ethics review committee in Ethiopia. Regulatory approval elicited questions about the need for purchase approval for the intervention product even though it was already publicly available. Approval at national level was delayed because this committee was disbanded in the week following submission of the GoLBet dossier, and was not reconvened for several months. Another reason for the delay at national level was loss of the study documents submitted for review, necessitating resubmission. Approval was eventually given at the start of the rainy season, so the decision was made to postpone screening until after the rainy season so as to maximise enrollment. A six-month extension to the project was requested and granted by the funding body.

## Results

### Listing and screening

Listing of patients started in the nine most accessible *kebeles* of Aneded district (*kebeles* 7–14 and 16, Fig. 2). In each of these *kebeles*, the two Health Extension Workers (local women with one year’s training in disease prevention and health promotion) were asked to list all people they knew to have leg swelling. The screening team (data collectors, data collector supervisors and the data manager) visited each potential participant at home and after oral informed consent, made a preliminary check against the inclusion and exclusion criteria that could be assessed verbally. Each person’s willingness to be considered for the trial was assessed, and those that wished to be considered were given an appointment for an enrollment visit at their nearest health post or health centre. Geographic Information System coordinates of the patient’s house were recorded, to be used (if the patient went forward to randomisation) to avoid randomisation of neighbours to different intervention groups. The screening team used a snowball approach, asking about other potential patients while in a given community, and visiting individuals suggested.

### Enrollment

Enrollment took place at government health posts and health centres. The enrollment team (data collectors, data collector supervisors, CPAs, CPA supervisors, the local safety monitor, the data manager, the trial coordinator and a laboratory technician) welcomed patients, gave group information about the trial and obtained individual informed consent. The consent form was signed by the participant if they were able to write. For those unable to write, they added their thumbprints and an independent witness signed to confirm the thumbprint and that all questions had been answered to the participant’s satisfaction, as agreed by the IRBs. They then made a full assessment of eligibility against the inclusion and exclusion criteria, including an immunochromatographic test. A unique study identification number was issued, and a photo taken for a trial identity card. The baseline Case Report Form was administered, and included socio-demographic information; frequency and duration of ADLA; Dermatology Life Quality Index; WHO-Disability Assessment Schedule; perceived stigma; and clinical signs including disease stage, foot and leg circumference, presence of mossy changes, wounds and entry lesions. Once this had been completed, the participant was informed that the result of randomization would be delivered to them at home and was given a copy of the information sheet and signed consent form to take home.

### Recruitment

**Fig. 3:**
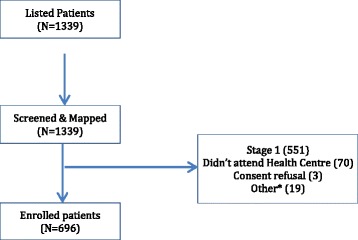
Overall listing, screening and enrollment flow

Given prevalence of podoconiosis in Amhara region of 3.9% and a district population over 100,000 (suggesting an adult population over 50,000), we originally assumed we would be able to recruit 680 patients for the trial using the nine most accessible *kebeles* in the district (numbers 7 to 14 and 16 in Fig. 2). However, in December 2014 and January 2015 we enrolled just 425 patients from these *kebeles*. After screening, rates of attendance at enrollment varied considerably from *kebele* to *kebele*, from 43% to over 90%. Feedback from field staff suggested this was predominantly due to potential participants being involved in the harvest and in compulsory local environmental protection campaigns. Very few (three) patients discussed the trial information and decided not to participate. However, in one *kebele*, misinformation spread by one individual alarmed patients. Acting on suggestions made during the Rapid Ethical Assessment [12] about quashing community rumours, the trial coordinator and data manager arranged an emergency *kebele* meeting to negotiate with gatekeepers and prevent further rumours being spread. The team made the decision to offer further enrollment sessions in the original 9 *kebeles* and to extend listing and screening to 9 of the 10 less-accessible *kebeles*. Using this approach, a further 271 patients were enrolled to reach the required sample size. The numbers enrolled are shown in Fig. 3.

### Randomisation

During a preparatory meeting with District Health Office personnel, concerns were raised about the use of randomization and the existence of a control group. The Rapid Ethical Assessment had covered these areas, and local parallels to randomization and delayed treatment to trial groups had been suggested. These included the random methods used to disburse sums from traditional savings systems such as ‘*Equb’* or decide whose turn it is to graze cattle, and comparisons of traditional and modern fertilizers used by agricultural development workers. These illustrations were used to explain the trial process at community meetings and with individual patients attending enrollment sessions.

## Conclusions

### Trial setting

Aneded district is a challenging setting for any research study, and this trial, which required 12 intervention visits and 4 data collection follow-ups for each patient, was demanding for participants, the trial team and those managing logistics. This district was chosen as being representative of districts into which treatment might be expanded in real life, and because no treatment services had been established within the district prior to the trial. Aneded district was an appropriate choice, given that pragmatic trials such as GoLBet are designed to test the effectiveness of an intervention in the real world [13]. When the real world of patients is a remote, low-resource setting such as Gojjam, carrying out a high quality trial to GCP standards will demand considerable commitment from the trial team. Field staff will need physical stamina, ingenuity and resilience. While clear schedules and timelines are essential, the trial team will also need to be adaptable and contingency funding may be necessary to cover additional transport costs, for example when roads became impassable or bridges were washed away.

### Regulatory requirements

Trial registration was completed in January 2014 within four months of confirmation of funding. Gaining ethical and regulatory approvals was more complex, partly because this had to be done consecutively rather than in parallel. Delays introduced by the ethical review process have previously been noted to be significant bottlenecks to research [7], and our experience confirms this. Developing ethical review capacity and streamlining review processes will be vital in overcoming delays like this in future. Even though regulatory requirements state that IRB members will make field visits to monitor trial progress and participant safety, these visits rarely happen in practice. We were aware of the value of sharing experience of this rare pragmatic trial with regulatory authorities, and so funded a two-member team from the National Research Ethics Review Committee to visit the study site.

### Case identification

We relied on lists compiled by Health Extension Workers to identify potential lymphoedema patients. Patients with podoconiosis often experience considerable stigmatisation [14, 15], so it is important that researchers approach patients through trusted members of the community such as health workers [16, 17]. Since podoconiosis has not until recently been included in health curricula, questions around the validity of identification by community workers exist. Earlier studies in Ethiopia have demonstrated that, in areas of high podoconiosis endemicity, health workers with very little training can identify patients accurately [18]. However, in areas of lower endemicity, such as North West Cameroon, the positive predictive value of community health worker identification is lower [19]. We used a wide case definition for the listing stage of the recruitment process, and then used a two-stage process (screening and then enrollment) to ensure that only eligible adult podoconiosis patients were invited to participate. Patients with leg swelling identified by Health Extension Workers overwhelmingly had podoconiosis; there were no positive immunochromatographic tests for lymphatic filariasis. The most common reason that patients screened were not enrolled was that they had stage 1 disease (551/1339 screened), that is, their leg swelling resolved overnight [20].

### Patient recruitment

Patient recruitment is frequently a challenge for RCTs, with UK funding bodies reporting less than one third of trials meeting their original targets [21]. A systematic review performed to identify interventions to improve recruitment included 45 trials [22]. Of these, only one trial had a single site in a low-income setting (the Philippines), and, unsurprisingly, few of the interventions were relevant to a remote rural setting like Gojjam. We experienced lower-than-anticipated recruitment in the nine most accessible *kebeles*, and used results from the Rapid Ethical Assessment [12] to mitigate this by quashing negative rumours and extending recruitment to more remote *kebeles*. The move to quash rumours might be construed as a departure from pragmatism, but as a team we considered the need to complete recruitment to outweigh efforts to preserve pragmatism during trial set-up. Rapid Ethical Assessment is increasingly used to ‘map’ the ethical terrain of a research setting prior to recruitment of participants [16, 17, 23, 24], and has been demonstrated to improve recruitment and retention rates in longitudinal studies [25]. We therefore strongly recommend the use of Rapid Ethical Assessment prior to complex studies including trials, in research-naïve settings.

### Data analysis

The Ethiopian calendar is a solar calendar similar to the Julian calendar. There are twelve months each of 30 days, and one month of 5 days (6 in a leap year). The Ethiopian calendar runs over 7 years behind the Gregorian (‘Western’) calendar, so neither years nor months nor days synchronize between the two calendars. Calendar issues regularly cause difficulties with longitudinal studies using Western-developed software. The GoLBeT team made the decision to collect and enter data using the Ethiopian calendar, and during analysis to use an algorithm to convert dates to the Gregorian calendar. This approach has been used by other research teams in Ethiopia to overcome date inconsistencies [26].

In order to conduct a high quality trial whose results are generalizable to the real world setting, a balance must often be struck between internal and external validity. Through examples that arose during GoLBeT, we demonstrate that this need not always represent a trade-off: it is possible to achieve high standards in both spheres, but this is likely to require additional time (for example, to conduct Rapid Ethical Assessment prior to the trial) and resources (for example, to ensure the same quality of follow up and data collection in the most remote settings).

## Abbreviations

ADLA: acute dermatolymphangioadenitis
CPA: Community Podoconiosis Agents
CRF: Case Report Form
GCP: Good Clinical Practice
GoLBeT: Gojjam Lymphoedema Best Practice Trial
IOCC: International Orthodox Christian Charities
IRB: Institutional Review Board
ISRCTN: International Standard Randomised Controlled Trial Number
KWTRP: KEMRI/Wellcome Trust Research Programme
RCT: Randomised Controlled Trial
SOP: Standard Operating Procedure
UK: United Kingdom
